# Unraveling the Functions of the Macroalgal Microbiome

**DOI:** 10.3389/fmicb.2015.01488

**Published:** 2016-01-05

**Authors:** Ravindra Pal Singh, C. R. K. Reddy

**Affiliations:** ^1^Laboratory of Microbial Technology, Department of Bioscience and Biotechnology, Graduate School, Faculty of Agriculture, Kyushu UniversityFukuoka, Japan; ^2^Seaweed Biology and Cultivation, Division of Marine Biotechnology and Ecology, Council of Scientific and Industrial Research–Central Salt and Marine Chemicals Research InstituteBhavnagar, India; ^3^Academy of Scientific and Innovative ResearchNew Delhi, India

**Keywords:** macroalgae, bacteria, functional species, cultivation, health

## Abstract

Macroalgae are a diverse group of photosynthetic eukaryotic lower organisms and offer indispensable ecosystem services toward sustainable productivity of rocky coastal areas. The earlier studies have mainly focused on elucidation of the roles of the epiphytic bacterial communities in the ecophysiology of the host macroalga. However, mutualistic interactions have become topic of current interest. It is evident from recent studies that a fraction of epiphytic bacterial communities can be categorized as “core microbial species”, suggesting an obligate association. Epiphytic bacterial communities have also been reported to protect macroalgal surfaces from biofouling microorganisms through production of biologically active metabolites. Because of their intrinsic roles in the host life cycle, the host in turn may provide necessary organic nutrients in order to woo pelagic microbial communities to settle on the host surfaces. However, the precise composition of microbiomes and their functional partnership with hosts are hardly understood. In contrast, the microbial studies associated with human skin and gut and plants have significantly advanced our knowledge on microbiome and their functional interactions with the host. This has led to manipulation of the microbial flora of the human gut and of agricultural plants for improving health and performance. Therefore, it is highly imperative to investigate the functional microbiome that is closely involved in the life cycles of the host macroalgae using high-throughput techniques (metagenomics and metatranscriptomics). The findings from such investigations would help in promoting health and productivity in macroalgal species through regulation of functionally active microbiome.

## Introduction

Most animals and plants rely on the subtle interactions of specific microorganisms for their successful sustenance. The emergence of high-throughput technologies has recently provided newer insights in understanding the complex interactions of microorganisms with diverse hosts such as the human gut, plant rhizosphere, and sponge microbiomes at an unprecedented depth (Turnbaugh et al., 2007; Berg and Smalla, 2009; Yang and Li, 2012). These microbiomes are highly diverse and are functionally connected with their eukaryotic hosts. Thus, these associations are now termed holobionts (Zilber-Rosenberg and Rosenberg, 2008). Particularly, microorganisms that colonize different parts of the human, coral, sponge, and plant bodies contain specific functional genes which are now regarded as secondary genomes (Siboni et al., 2008; Grice et al., 2009; Lebeis et al., 2012; Bulgarelli et al., 2013). The application of metagenomics has provided significant new information with respect to interaction of phyllospheric, rhizospheric, and endospheric microbiomes of higher plants. The prevailing environmental conditions have also been considered to be potential drivers for shaping and determining the host microbiome (Turner et al., 2013). Secondary metabolites (flavonoids) have also been shown as key determinants for formation of plant-specific rhizospheric microbiomes (Weston and Mathesius, 2013). Furthermore, investigation of the human gut and the plant rhizospheric microbiomes has provided new means for modulation of specific microbial communities in order to reduce disease incidence particularly in the former host (Andrews, 1992; Bloemberg and Lugtenberg, 2001), and chemical inputs (Adesemoye et al., 2009) and emission of greenhouse gases (Singh et al., 2010) in the latter to accelerate agricultural productivity (Bakker et al., 2012). Similarly, macroalgal growth and development are shown to depend on associated microorganisms, particularly bacterial communities (Singh and Reddy, 2014; Wichard et al., 2015). Despite the publishing of several interesting findings regarding the interaction of macroalgae and their bacterial communities in last decade, the functional diversity and connectivity of these communities with the host is yet to be tapped.

In regard to the functional connection between macroalgae and their associated bacteria, it has been reported that bacterial cells chemically communicate with the host and symbiotically assist in the processes of growth, morphogenesis, and reproduction (Chisholm et al., 1996; Joint et al., 2002; Matsuo et al., 2005) by modulating the abiotic and biotic interactions of the association (Wahl et al., 2012). Functional understanding of the chemical signaling in the macroalgal-bacterial interaction is also limited as compared to higher plants (Hartmann et al., 2014), despite the presence of ecophysiological evidences since a long time (Provasoli and Pintner, 1980). Therefore, in this article, we emphasize the need for re-investigating the existing knowledge of bacterial assistance in the life cycle of macroalgae using high-throughput technologies to understand the functions of the macroalgal microbiome and host responses.

## Macroalgae as Important Marine Habitat Formers and a Growing Economic Resource

The macroalgal canopy at intertidal regions is important for safeguarding intertidal ecosystems. For example, removal of macroalgal species can modify the local habitat and alter the impact of spatial complexity on the surrounding benthic species (Tait and Schiel, 2011). It has also been observed that declining intertidal macroalgal species leads to a loss of associated species that rely on the established algal canopy (Komatsu et al., 2014). Macroalgae also have immense commercial value. They are rich in minerals and essential trace elements required for human consumption as well as important sources of raw materials for fertilizers, hydrocolloids, and biofuel industries (Kumari et al., 2010; Baghel et al., 2015). Growing energy demands and rapidly depleting fossil fuel reserves have collectively stimulated the search for sustainable alternative bioenergy sources and supplies (Hahn-Hägerdal et al., 2006). In this context, macroalgae have recently been recognized as a potential source of renewable biofuel and bioenergy (Baghel et al., 2015). Due to industrial demands, they are commercially cultivated in a number of countries (Loureiro et al., 2015). Several industries provide a wide variety of products derived from macroalgae that have an estimated total annual value of US$ 5.5–6 billion. Additionally, diverse food products processed from macroalgal raw materials have a contributed value of over US$ 5 billion (FAO, 2014). Thus, increasing the gross production of industrially important macroalgal species is required for fulfilling human demands. For this purpose, identifying the functionally active microbial species associated with them could accelerate their production through appropriate modulation.

## Macroalgal Microbiomes Assist in Host Life Cycle

Epiphytic and endophytic bacterial communities have been extensively studied in the context of their phylogenetic composition and variability on or in macroalgae (Staufenberger et al., 2008; Lachnit et al., 2011; Hollants et al., 2013). These studies have used morphological characterization, *in situ* hybridization, electrophoresis, DNA finger printing, pyrosequencing, and metagenomic approaches (Tujula et al., 2010; Burke et al., 2011b; Hollants et al., 2011, 2013; Lachnit et al., 2011; Bengtsson et al., 2012; Bondoso et al., 2014). These findings suggest that bacterial communities belonging to the phyla *Proteobacteria* and *Firmicutes* are generally the most abundant epiphytic bacteria associated with macroalgal hosts.

Studies from previous decades have also revealed the role of epiphytic bacteria in the ecophysiology of macroalgal hosts. The importance of bacteria in macroalgal research began with the study by Provasoli and Pintner (1953), who reported that plant growth regulators (indol-3-acetic acid) regulate growth and morphogenesis in *Ulva* species. Provasoli (1958) reported that an axenic culture of *Ulva* did not develop into normal foliose morphology and showed polymorphic behavior. Later, it was observed that *Ulva* species retained normal foliose structures when their cultures were inoculated with specific bacterial communities (Tatewaki et al., 1983). In fact, this observation was attributed to thallusin, a bacterial compound obtained from the specific bacterial strain YM2-23 which was found to restore normal thallus morphology (Matsuo et al., 2005). Additionally, some bacterial species produce regulatory compounds resembling cytokinin (from *Roseobacter*, *Sulfitobacter*, and *Halomonas*) and auxin (from *Cytophaga*) that assist in the differentiation of *U. mutabilis* (Spoerner et al., 2012). Interestingly, unidentified metabolites from Gram-positive bacteria have also been found to induce morphogenesis in *Ulva* species (Marshall et al., 2006; Singh et al., 2011). These findings suggest the possibility that these unidentified compounds may have broad-spectrum activities with possible host benefits. Thus, such potential compounds should be identified by further studies.

Discovery of the quorum sensing (QS) system as a mechanism of cooperative behavior in bacteria (Fuqua et al., 1994) was yet another revolutionary subject of interest. QS has been defined as the cell-cell communication system that exists between same and different bacterial populations and depends on the threshold concentration of cells (Rutherford and Bassler, 2012). A group of QS signaling compounds, *N*-acyl homoserine lactones (AHLs) secreted by Gram-negative bacteria has gained significant attention in higher plants due to several phenomena of plant life cycles being controlled by AHLs via inter-kingdom communication system (Venturi and Fuqua, 2013; Hartmann et al., 2014). Interestingly, macroalgae are also involved in inter-kingdom communication through AHLs, and earlier studies have indeed demonstrated that AHLs promote the settlement of zoospores in green macroalgae (Joint et al., 2002) and liberation of carpospores in some red macroalgae (Weinberger et al., 2007; Singh et al., 2015). Thus, it is essential to determine the molecular mechanisms involved in these reported chemical cues in macroalgal hosts through further studies.

Additionally, many fundamental questions regarding chemical signaling systems and the interactive functions of macroalgal-bacterial interaction remain to be resolved for a better understanding of the macroalgal microbiome. For examples, two aspects of the bacterial role in macroalgal life cycles have been well studied, (a) their role in host reproduction and growth and (b) the induction of morphogenesis in green macroalgae. Despite these, we do not know how these phenomena are regulated by signaling cascades or the kinds of secondary molecules involved and how these phenomena are regulated at the DNA, RNA, and protein levels of this association. Thus, we would like to suggest that future studies should emphasize on meta-omics technologies to understand the macroalgal microbiome in depth.

## Next-Generation Tools for Unveiling Functional Bacterial Genomics of Macroalgal Microbiome

**FIGURE 1:**
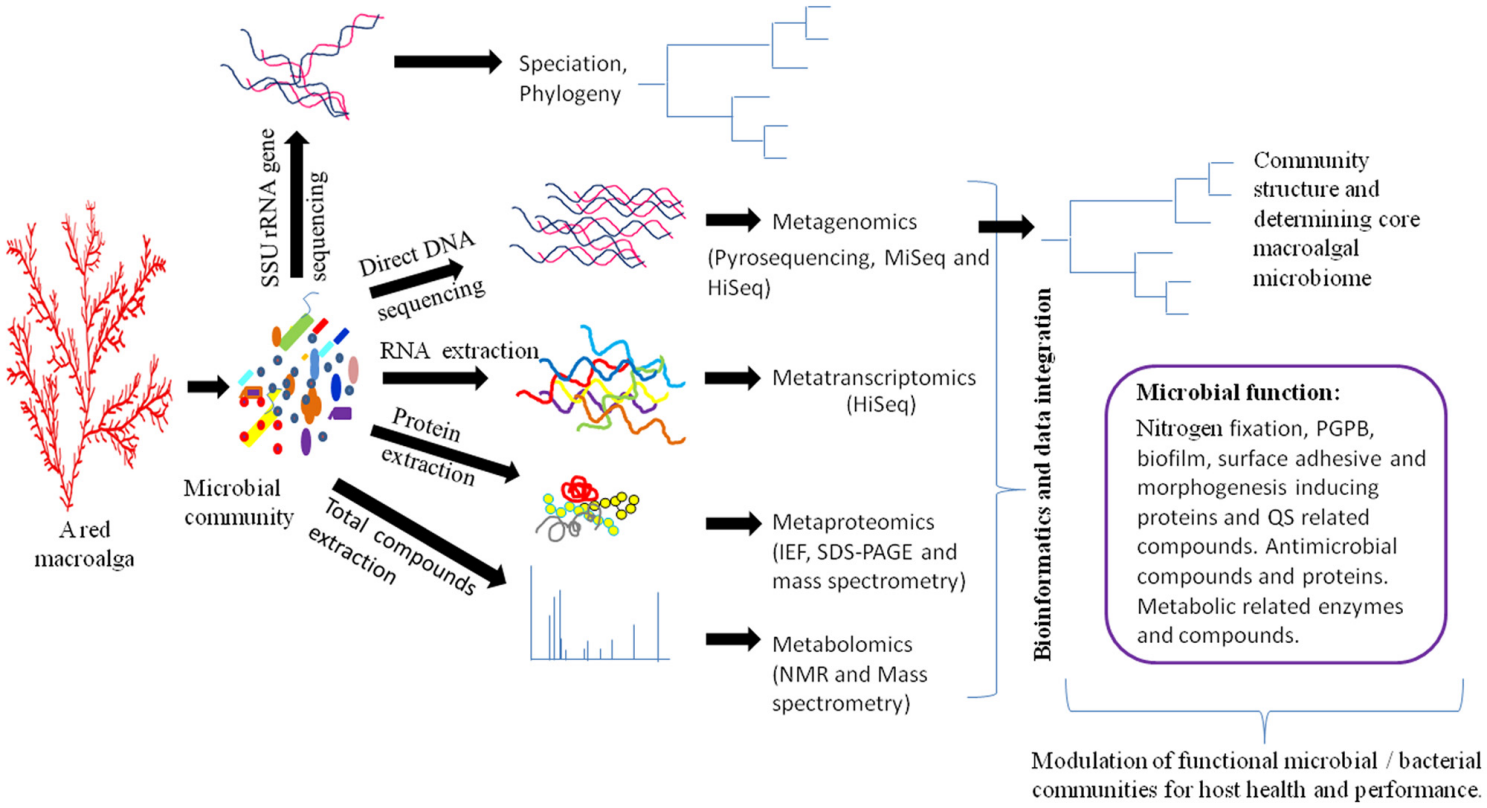
**Schematic diagram for elucidating the functions of the macroalgal microbiome.** Different meta-omics techniques can be used to identify active functional microbial/bacterial communities. Subsequently, these communities can be used to modulate macroalgal growth and health. Metagenomics and metatranscriptomics analyses can be obtained by Mi-Seq, Hi-Seq, 454 GS FLX SOLiDv4, and Sanger 3730xl sequencing methods (Liu et al., 2012). Functional annotation of metagenomic data can be determined by MG-RAST, IMG/M, METAREP, CAMERA, and MEGAN4 softwares (Kim et al., 2013). Metaproteomics of microbial data can be analyzed by isoelectric focusing (IEF), sodium dodecyl sulfate polyacrylamide gel electrophoresis (SDS-PAGE), matrix-assisted laser desorption/ionization time-of-flight mass spectrometry (MALDI-TOF-MS), and liquid chromatography–mass spectrometry (LC-MS/MS) (Kan et al., 2005). The metabolome of microbial communities can be identified by nuclear magnetic resonance spectroscopy and diverse mass spectrometry methods including MALDI-TOF and LC-MS/MS (Lee et al., 2012a,b). PGPB- plant growth-producing bacteria, QS- quorum sensing.

Next-generation sequencing (NGS) technologies (metagenomics and metatranscriptomics) have had a dramatic impact on the field of microbial genomic research through stipulation of low cost and high-throughput sequencing systems such as Hi-Seq and Mi-Seq (Caporaso et al., 2012). These NGS technologies follow high-throughput and powerful analytical methods called “metabolomics” to monitor the actual physiological state of microbial communities. Another technique, called “metaproteomics,” determines the actual post-transcriptionally regulated and translated microbial proteins under a given condition in addition to unveiling the active molecular interactions of the microbiome (**Figure 1**). The technical details of these meta-omics technologies have been comprehensively reviewed elsewhere (Mardis, 2008; Metzker, 2010; Schadt et al., 2011; Tang, 2011; Kolmeder and de Vos, 2014) and are not discussed here. Instead, this section briefly summarizes these technologies in context of their potential applications in functional bacterial genomics of the macroalgal microbiome with examples from human and plant microbiomes.

Metagenomics refers to the culture-independent method that is used to explore genetic diversity, population structure, and interactions of microbial communities in their ecosystems. This approach has identified at least 5000 novel, out of 19,000 functional gene clusters annotated from human microbiome projects such as the MetaHIT (http://www.metahit.eu). Many of the genes comprised in the core metagenome are essential for the healthy and proper functioning of the intestinal ecosystem (Qin et al., 2010). Knowledge of this association allows the development of a new range of diagnostic techniques and therapeutics to modulate, enhance, and maintain intestinal homeostasis and thus promote intestinal activities (Turnbaugh et al., 2006; Tana et al., 2010; Kostic et al., 2012) and general health (Wang et al., 2011). Similarly, metagenomics has provided significant knowledge of plant microbiomes (Turner et al., 2013) and has been applied to increase plant health and growth as aforementioned and reported by Mendes et al. (2011). Macroalgae associated bacterial communities are paramount for the host life cycle; however, functional information from culture independent bacterial communities is poorly understood, especially from red and brown macroalgae (Singh and Reddy, 2014). A previous metagenomic study by Burke et al. (2011a) identified several important clusters of orthologous groups (COGs) in bacterial communities associated with *U. australis* that were related to the algal host environment, such as degradation of host secreted metabolites, overcoming host oxidative burst defense mechanisms, storage of heavy metals, and desiccation. Additionally, several cluster homologues to COG0642 (histidine kinase) that are involved in nitrate reduction, motility, QS system, osmoregulation, cell differentiation, plant virulence, and related to defense (involved in host defense by inhibiting growth of other bacteria present in pelagic form) were found. Abundance of these genes in associated bacterial communities indicated that these mediated interaction with the host and other prokaryotic and eukaryotic communities (Burke et al., 2011a). However, it is not known whether these functional bacterial clusters are actively involved in macroalgal-bacterial interaction or whether these clusters can be used for modulating the macroalgal microbiome for defense and growth. Thus, it is important to perform other meta-omics studies (as mentioned below) parallel to metagenomics in order to specify the physiological status of specific functional bacterial communities.

Metatranscriptomics is recognized as a far more accurate method of measuring the levels of transcripts induced by any compound as compared to other methods (Wang et al., 2009). Metatranscriptomic analysis can show a substantial fraction of differentially regulated microbial transcripts from a microbiome sample (Bikel et al., 2015). Metatranscriptomics can be applied to associated bacterial communities to understand the precise level of functional connection of the host microbiome. Metagenomics in combination with metatranscriptomics has unveiled several interesting facts. For example, specific induction of microbial genes in the gut microbiome has been observed in response to host targeted exposure of xenobiotics (Ursell and Knight, 2013). Recently, transcriptomics analysis has been applied to the red macroalga (*Laurencia dendroidea*) and their associated microbiome (de Oliveira et al., 2012). Transcripts of associated bacterial communities were highly related to glycolysis, lipid, and polysaccharide breakdown indicating that associated bacteria rely on carbohydrate sources secreted by the host for energy. Amino acid metabolism related transcripts suggested that compounds relevant to nitrogen fixation are exchanged between the host and bacteria. The study also found transcripts related to cell motility and chemotaxis for recognizing the macroalgal surface and establishment of biofilm as well as infection related transcripts particularly of vanadium-dependent bromoperoxidases in associated bacterial communities. RNA transcripts related to oxidative stress mechanisms indicated that the macroalgal associated microbiome utilized aerobic metabolism and also minimized the oxidative burst mechanism in macroalgae (Weinberger, 2007). Elevated transcripts of the QS system indicated that the presence of significant intra- and inter-kingdom communication, which is scarcely understood in comparison with higher plant systems (Hartmann et al., 2014). Many transcripts found in the bacterial communities of *L. dendroidea* are congruent with COGs of metagenomic clusters found in bacterial communities of *U. australis*, indicating that integrated chemical interaction occurs in this association. Thus, performing a metatranscriptomics study in the context of identifying states of metabolism exchange will be helpful for an in depth understanding of the macroalgal microbiome.

Metaproteomics is the study of all proteins directly recovered from complex microbial communities. This analysis provides information to gain insights into the functioning of microbial components in the host, beyond the limitation of nucleic acid-based methods (Wilmes and Bond, 2006; Maron et al., 2007). DNA sequence data comprises of many genes with unknown functions and involves a high abundance of unknown functional systems (Tringe et al., 2005). Hence, metaproteomics might prove invaluable for their identification and defining proper functions (Wilmes and Bond, 2006). Complete scanning and characterization of the metaproteome of associated microbiomes is expected to provide data linking the genetic and functional diversity in connection with the specific host (Maron et al., 2007). Additionally, it provides direct evidence for the expressed and functional genes during host-microbial interaction. For example the study by Verberkmoes et al. (2009) employed a shotgun mass spectrometry-based metaproteomics approach and found that more proteins of microbial communities related to translation, energy production, and carbohydrate metabolism were observed than those predicted from the metagenomics approaches. Metaproteogenomics of microbial communities of the rice plant revealed that the functional potential of microbial communities depends on their localization, i.e., phyllosphere versus rhizosphere (Knief et al., 2012). The physiological traits of transport processes and stress responses were more prominent in phyllospheric samples whereas dinitrogenase reductase was solely identified in the rhizospheric microbiome, despite the presence of *nifH* genes in diverse phyllospheric bacterial communities. The functional interaction of the macroalgal microbiome is very poorly understood in view of metaproteomics and metaproteogenomics, despite its complex dynamic functional connection. Particularly, this analysis will help to identify the proteins of associated microbial communities that are involved in morphogenesis and growth of the host macroalga as well as the proteins that are important for development of the microbiome on the host surface. It will also provide an insight into the pathogenicity (possibly via protease and polysaccharide-degrading enzymes) of bacterial pathogens of the macroalga since true pathogens are already known (Vairappan et al., 2001; Zozaya-Valdes et al., 2015).

The metabolome refers to the complete set of small-molecules produced by an organism and are a representation of the metabolic pathways and networks of the genome. Microbial metabolites can be determined using a number of different technologies including nuclear magnetic resonance spectroscopy and diverse mass spectrometry (Lee et al., 2012a,b). Metabolomics is the study of the metabolome that can potentially provide a perfect portrait of the definite physiological state of a specific microbiome because the intermediates of various biochemical reactions play a vital role in connecting different pathways that operate in active microbial communities (Tyson et al., 2004; Garcia et al., 2008; Nicholson and Lindon, 2008; Tang, 2011; Lu et al., 2014). Thus, microbial metabolomics is an important component of systems biology that facilitates the understanding of integrated microbial functions. Additionally, they are easy to manipulate through pre- and probiotics and have crucial functions in health and growth of different eukaryotic organisms including humans and macroalgae. For example, there are approximately 10^14^ bacterial cells present in the human gut belonging to about 1,000 bacterial species that are known to have a direct bearing on individual health (Nicholson et al., 2005; Gill et al., 2006; Kassinen et al., 2007; Atarashi et al., 2011). These microorganisms produce several metabolites (such as butyrate) that are involved in defense against pathogens, maintaining homeostasis, inducing cellular differentiation in the immune system (CD4^+^Foxp3^+^ regulatory T cells), secretion of essential anti-inflammatory molecules (interleukin-10 and inducible T-cell co-stimulator), and renewal of gut epithelial cells (Turnbaugh et al., 2007; Atarashi et al., 2011, 2013). The dynamic metabolic flux of plant rhizosphere and bacterial communities is extensively studied in the context of rhizobial interaction with their hosts (Venturi and Fuqua, 2013). Inter-kingdom signaling through AHLs and plant roots has been significantly demonstrated in recent times wherein AHLs produced by bacterial communities were found to assist in plant growth, development, and performance (Hartmann et al., 2014). Similarly, dynamic interactions of controlled metabolism exist between the macroalgal microbiome and co-metabolism, that are contributed by both bacterial communities and the host (Dittami et al., 2014). Notably, few chemical compounds have been identified from associated bacterial communities that determine growth and morphology of *Ulva* (Wichard et al., 2015). However, several unknown bacterial compounds have been observed to induce morphogenesis in macroalgal hosts (Marshall et al., 2006; Singh et al., 2011). Therefore, microbial metabolomics should be included in macroalgal research for studying the spatiotemporal dynamics of metabolite production in bacterial communities and for identifying potential compounds.

Several bioinformatics softwares and statistical tools are being used to analyze a large amount of raw data obtained from NGS experiments. The information of these tools with brief descriptions is available at http://bioinformaticssoftwareandtools.co.in/ngs.php. The data generated from these meta-omics technologies will undoubtedly revolutionize our understanding of the macroalgal microbiome under given conditions, particularly in identifying several unknown proteins and active metabolic compounds and the defining regulatory processes of this association. The integrated information of these techniques will allow construction of detailed, high-resolution regulatory maps of biological function for macroalgal-bacterial interaction (**Figure 1**). The information obtained from these meta-omics technologies will be underpinned to exploit them in the future with respect to development of potential macroalgal probiotics as mentioned below.

## Modulating Macroalgal Microbiome for Host Health and Performance

The development of NGS was a revolutionary step in microbial ecology that has provided newer insights in understanding the complex host-microbe interactions, especially in the context of non-cultivable microorganisms and finding beneficial microbial species of the host. An artificial consortium of bacterial species representing the beneficial gut microbiome of a healthy person when transferred into the gut of infected mice results in re-establishment of normal microflora and leads to mitigation of the gastro-intestinal diseased condition (Petrof et al., 2013; Narushima et al., 2014). In agriculture, successful plant disease management has been achieved through transferring active beneficial microbiomes by mixing disease suppressive soils with disease conducive soils (Mendes et al., 2011). In another study, shifting of the soil microbiome was achieved through soil solutions in which the modulated microbiome alleviated drought stress in *Arabidopsis thaliana* (Zolla et al., 2013). These findings suggest that beneficial microbial species would been favored during early development of the host by certain selective pressures, probably on the basis of prevailing environmental conditions and then participated in further development of the host microbiome.

Successful modulation of beneficial microbial/bacterial species of the human gut and plants has provided new avenues to study the macroalgal microbiome from the context of their interactions and functional relationship with hosts. Implementation of a variety of meta-omics technologies on microbial communities of macroalgae will identify a specific functionally active bacterial species (FABS) and establish their functional relationship with the hosts. Such FABS will serve for improving health of the host by suppressing growth of pathogen(s) and enhance performance by assisting the host physiology. Thus, in the future, macroalgal functional microbiomes will have a greater importance for macroalgal cultivation to continuously supply raw materials for diverse industries and to fulfill human demand. For example, FABS can be identified from healthy individual(s) and be applied to diseased plantlets in order to suppress the growth of pathogen(s) or in case of commercial cultivation, germling fronds may first be treated with the FABS identified in the laboratory and then transferred either into controlled farms or in open seas for improving growth performance.

## Author Contributions

RS and CR had designed the outlines of the work. Then, RS has drafted the initial version of the work and CR has substantially improved the quality of the work as well as approved the final version to be published.

## Conflict of Interest Statement

The authors declare that the research was conducted in the absence of any commercial or financial relationships that could be construed as a potential conflict of interest.
